# The impact of surgical guide design and bone quality on heat generation during pilot implant site preparation: an *in vitro* study

**DOI:** 10.1186/s12903-023-02961-9

**Published:** 2023-05-10

**Authors:** Eman Abuhajar, Nesreen A. Salim, Malik Sallam, Fadi Jarab, Julian D. Satterthwaite

**Affiliations:** 1 Faculty of Medicine and Dentistry and Oral Surgery, Honorary position at Tripoli University, University of Tripoli, Tripoli, Libya; 2 Prosthodontic department, School of Dentistry, consultant in fixed and removable prosthodontics, The University of Jordan, Jordan University Hospital, Amman, Jordan; 3 Department of Pathology, Microbiology and Forensic Medicine, School of Medicine, The University of Jordan, Amman, 11942 Jordan; 4 Department of Clinical Laboratories and Forensic Medicine, Jordan University Hospital, Amman, 11942 Jordan; 5 Department of Oral Medicine and Oral Surgery, Faculty of Dentistry, University of Science and Technology, Irbid, Jordan; 6 Restorative Dentistry, Division of Dentistry, School of Medical Sciences, University of Manchester, Oxford Road, Manchester, M13 9PL UK

**Keywords:** Dental implants, Osteotomy, Non-limiting template, Guided template, Surgical guides, Temperature elevation

## Abstract

**Background:**

Surgical guides restrict the flow of cooling agent to osteotomy site, which will lead to a temperature rise that provokes tissue injury. Few studies compared differences in the temperature changes between non-limiting ‘conventional’ and limiting ‘guided’ surgical guides during implant site preparation. The objective of this study was to investigate the difference in temperature changes during bone drilling for implant placement using non-limiting and limiting surgical guides at cortical and cancellous bone levels.

**Methods:**

Forty-four bovine rib samples were used for implant bed preparation in this study with a minimum thickness of 11 mm was chosen for the ribs. The bone was stored in a freezer at 10 °C until it was used. On the day of the study, the bone was defrosted and soaked in water at 21 °C for three hours before embarking on drilling to make sure each sample was at the same temperature when tested. Forty-four bone specimens were prepared and randomly allocated to receive either a limiting or a non-limiting surgical guides (22 for each group). The osteotomy site was prepared by one operator following the manufacturer’s instructions, using limiting and non-limiting surgical guides. Temperature changes were recorded during implant bed preparation using thermocouples that fit into 7 mm-horizontal channels at two different depths (Coronally) and (Apically) at 1 mm distance from the osteotomy site. The data were tested for homogeneity of variances using Levene’s test, then data were analyzed using an Independent sample t-test and the significance level was set at P ≤ 0.05.

**Results:**

The mean temperature rise for all samples was 0.55 °C. The mean temperature rises for the limiting and non-limiting surgical guides were 0.80 °C and 0.33 °C respectively. There was a statistically significant difference in temperature rise between the limiting and non-limiting surgical guides (P = 0.008). In relation to position of temperature recording (coronal vs. apical), there was no significant difference (P > 0.05). No significant difference was noted between the two groups at cancellous bone level (P = 0.68), but the difference was significant at cortical bone level (P = 0.036).

**Conclusion:**

Limiting surgical guides showed higher readings than non-limiting. However, for both techniques, temperature rise was not significant clinically and within a safe range.

## Background

Implant dentistry is a successful option for replacing missing teeth [1–3]. The success of implants depends on the interaction between the implant itself in terms of the material, surface treatment and bone quality and quantity, as well as the ability to heal and the implant bed preparation, including drilling of the bone using twist drills [4]. Drilling is clearly the most important procedure during implant site preparation, as it determines the outcome of osseointegration, but causes mechanical and thermal injury to the bone [5]. Drilling for more than one minute at temperatures over 47 °C (i.e. a 10 °C rise) induces local osteonecrosis and inhibits osseointegration and in order to avoid thermal injury these drills together with the surgical site are universally water-cooled [5].

Surgical guides have evolved to facilitate precise implant placement, by transferring the preoperative plan to the surgical field in a correct position that fulfils the functional and aesthetic requirements [5, 6]. Surgical guides, either non-limiting or limiting, will restrict the flow of the cooling agent to the osteotomy site, which will lead to a temperature rise that provokes tissue injury if exceeding the maximum threshold [6, 7]. Surgical guides they can be fabricated either manually [6], or with aid of CAD/CAM technology [6, 7]. Advances in patient imaging and planning allow guides to be constructed that limit the orientation of the twist drill to the required angle [8].

The amount of heat generated with limiting surgical guide was considered to be more when compared to free hand placement [9, 10]. However, it has been reported that a limiting method creates intraosseous temperatures that are still within the safe range [10–13]. In vitro studies have shown that heat has a negative effect on future bone healing and that bone can sustain a crucial temperature threshold value of 47 °C without necrotic disintegration [14, 15]. Further, it has been suggested that even late complications resulting in implant failure are related to heat generation at the drilling site [16].

.

The different types and accuracy of surgical guides has been well reported in the literature [7]. These surgical guides, either non-limiting or limiting, will restrict the flow of the cooling agent to the osteotomy site, which will lead to a temperature rise that provokes tissue injury if exceeding the maximum threshold [6]. Surgical guides may be broadly described as being non-limiting (conventional) or limiting (either partially or fully). A non-limiting guide is less restrictive than a limiting guide, and typically allows for greater access to the surgical site, both for direct vision of the surgical field, and also improved contact of the irrigation fluid with the implant drill. Fully limiting guides typically allow for precise positioning and angulation of the osteotomy via a hollow cylinder or ‘guide tube’ and this tube restricts the flow of water coolant onto the drill and surgical site [5, 13], and it has been demonstrated that the use of such a surgical guide for implant placement generates more heat than a non-limiting surgical template [9].

There are relatively few studies comparing non-limiting and limiting surgical guides, so differences in the temperature changes between them during implant site preparation are not clear. The objective of this experimental in vitro study was to evaluate the impact of the surgical guide design on heat generation at the cortical and cancellous areas at the osteotomy site. The null hypothesis was that there is no impact of the surgical guide design (non-limiting and limiting) on heat generation at the cortical and cancellous areas at the osteotomy site.

## Materials and methods

### Study design

**Fig. 1:**
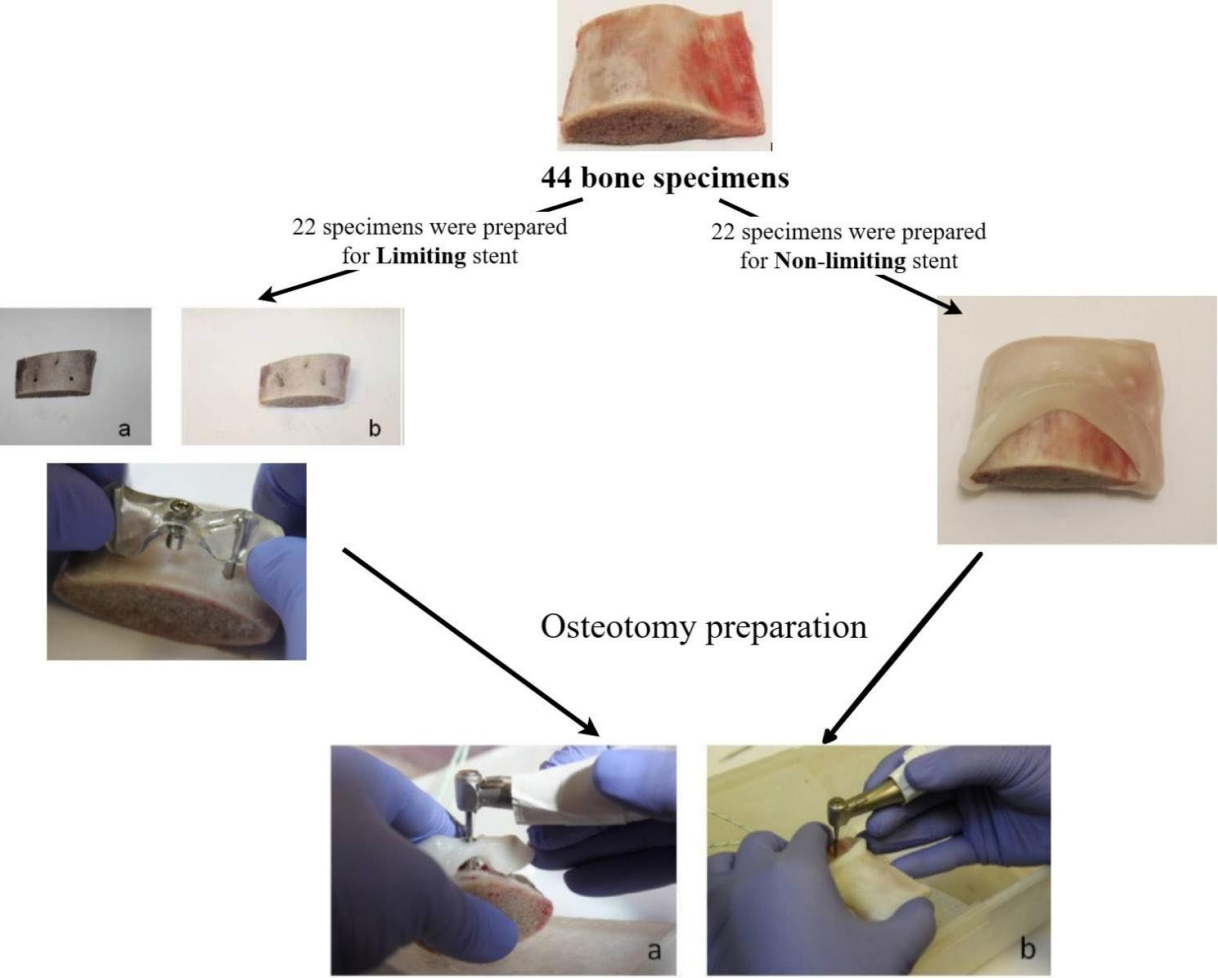
Flow chart showing the main steps in specimens’ preparation for both limiting and non-limiting stents. Bone specimen with holes created to receive metal rods (a) bone specimen with metal rods (b)

The effect of different surgical guide designs on temperature changes during osteotomy in vitro was investigated in this experimental study. Forty-four bovine rib samples were used for implant bed preparation; twenty two samples for limiting surgical guides; and twenty-two samples for non-limiting surgical guides. Temperature changes were recorded using thermocouples at two different depths (Coronally) and (Apically) at 1 mm distance from the osteotomy site **(**Fig. 1**)**. Temperature measurements were recorded throughout the whole osteotomy procedure.

### Sample size calculation

A pilot study was conducted on ten samples of bovine bone to gain familiarity with different surgical guide types and fabrication techniques, as well as testing the efficiency of the coolant system and surgical motor used, and to carry out a sample size calculation. The bone samples were used for implant bed preparation; five samples for limiting surgical guide; and five samples for non-limiting surgical guide. Temperature changes were recorded using thermocouples at two different depths (Coronally) and (Apically) at 1 mm distance from the osteotomy site **(**Fig. 1**)**. Temperature measurements were recorded throughout the whole osteotomy procedure.

The data collected from the pilot study was analyzed using SPSS® (Ver. 20; SPSS Inc, Chicago, IL) software for Windows in order to calculate the sample size. A sample size calculation showed that a group size of twenty-two was sufficient to identify a clinically relevant difference (of ten degrees) between two groups assuming a standard deviation of 11.8 with a power of 80% at a significance level of 5%.

### Bone specimen preparation

Bovine bone ribs were obtained from local butcher with availability of good cortical and cancellous layers, and a minimum thickness of 11 mm were chosen for the study, this was standardized by measurement. The mass flesh and cartilage was removed by a knife. After that, each rib was cut into smaller blocks. The bone was stored in a freezer at 10 °C until it was used. On the day of the study, the bone was defrosted and soaked in water at 21^ο^C for three hours before embarking on drilling to make sure each sample was at the same temperature when tested. The bone was kept moist all the time until it was tested. Forty-four bone specimens were prepared and randomly allocated to receive either a limiting or a non-limiting surgical guide.

**Fig. 2:**
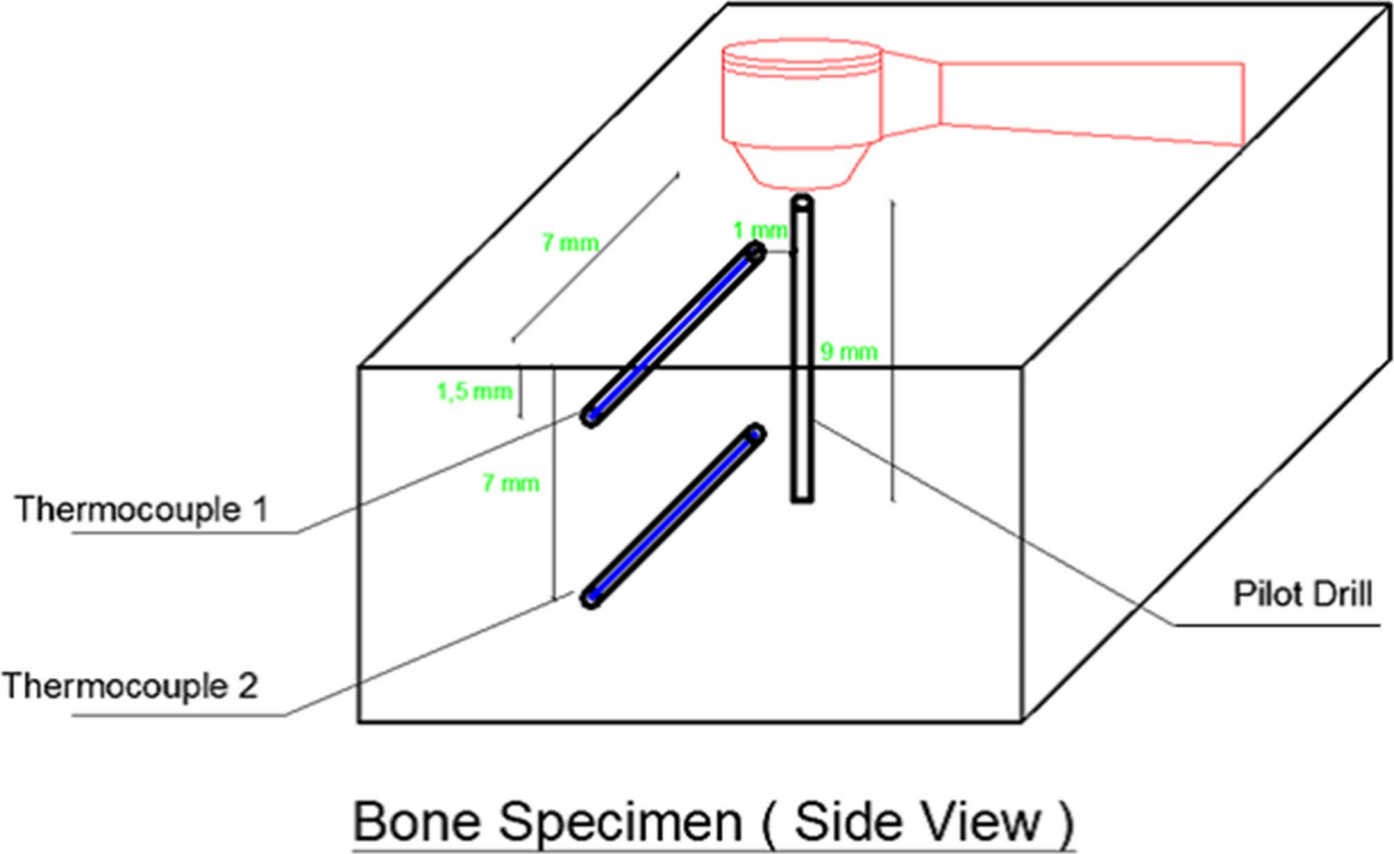
Diagram showing bone specimen with thermocouples and pilot drill. Two channels to accommodate thermocouples later were prepared at 1 mm distance from the osteotomy site

Firstly, the planned osteotomy site was decided and marked according to the quality of bone in terms of even surface and proper length. Then, two marks were measured and marked on the bone in a horizontal direction. These two points were planned to be at 1 mm distance from the osteotomy site. After that, the previously marked points were drilled with a round bur with a rubber stopper attached to it to standardize the distance to which the bur will travel at 7 mm in order to create channels to accommodate thermocouples later on **(**Fig. 2**)**.

The thermocouples were positioned in horizontal canal, at two different positions, which are **(**Fig. 2**)**:

- Coronally, in cortical bone 1.5 mm deep intraosseously.
- Apically, in cancellous bone 7 mm deep intraosseously.

### Surgical guide fabrication

#### 1- Non-limiting surgical guide

Thermoplastic beads (Nociceptive Trigeminal Inhibition Tension Suppression System, Inc, Indiana, USA) were heated in water and mixed together in order to have a homogenous ball of material. After that, the material was adapted on the bone specimen while still translucent. Then a window was cut out with a blade leaving some of the material to form a short wall on one side of the window, replicating a non-limiting surgical guide **(**Fig. 1**)**.

#### 2- Limiting surgical guide

A limiting surgical guide (thermoplastic drill templates set single tooth, Lot CE895, Straumann, Basel, Switzerland) was fabricated following the manufacturer’s instructions. In order to support the guide, prior to temperature assessment/osteotomy two holes were drilled into the rib sample (using a 2.8 mm diameter drill to a length of 7 mm Fig. 1**)**; the distance between the two holes was 25 mm center to center. Then metal rods supplied with the surgical guide kit (length 20 mm and diameter 2.3 mm) were placed in the holes to support the surgical guide at both ends. The thermoplastic surgical guide was softened in hot water and adapted to slide over the metal rods **(**Fig. 1**)**. The surgical guide was placed leaving 4-5 mm of space between the surgical guide and the bone surface to resemble the clinical situation. This distance was standardized by adapting the surgical guide while soft to the metal rod at a certain marked point. This was done for all limiting surgical guides used in the experiment.

### Osteotomy preparation

**Fig. 3:**
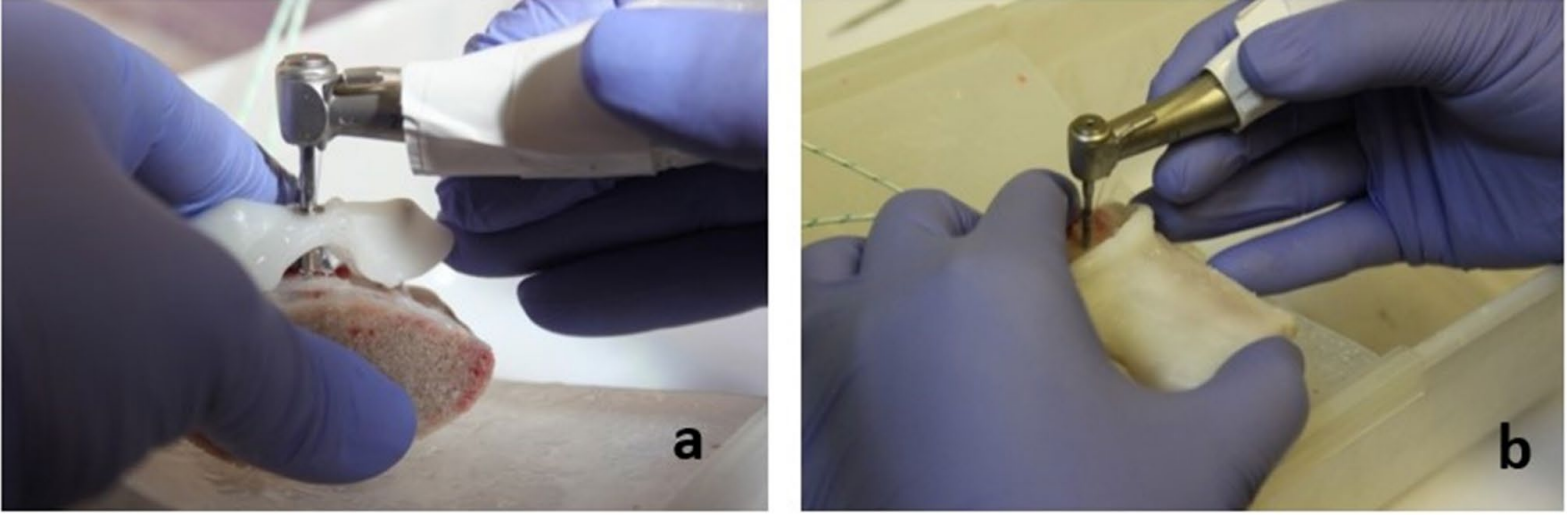
Osteotomy preparation for limiting stent (a) non-limiting stent (b)

The same operator performed all osteotomies **(**Fig. 3**)**, and one osteotomy per block was completed. A surgical motor and hand piece was used, which was calibrated before each osteotomy following the manufacturer’s instructions (NSK Surgical Pro series surgical unit (NSK, Stevenage, UK)). The speed used for drilling was 800 rpm. First, the osteotomy site was marked on the bone with a round stainless steel bur (size Ø1.4 mm, Lot CR171, Straumann), following that a short pilot drill (size Ø2.2 mm, Lots CP624, EG848, Straumann) was used to prepare the osteotomy site (as recommended by the thermoplastic limiting surgical guide manufacturer). The pilot drill was compatible with the guide sleeve inner diameter which was Ø2.3 m. All osteotomies were prepared to a depth of 9 mm due to the limited thickness of the bovine ribs. All drills were used ten times prior to disposal. The time taken to change the drills during the osteotomy preparation was standardized at twenty-two seconds by using a stopwatch.

### Irrigation

Normal saline (0.9% sodium chloride 100ml, Fresenius Kabi Ltd, Runcorn, UK) at room temperature was used for irrigation. External irrigation was used in this study. The irrigant flow rate was 26.67ml/minute.

### Temperature measurement

Temperature was recorded using a thermocouple type K welded tip (Pico Technology Ltd, UK), which was placed in the prepared channels in the bone specimen **(**Fig. 2**)**, and immobilized in place by aid of thermal adhesive paste (Arctic silver Alumina thermal adhesive paste 2.5gm part A and part B, Shiny hardware Ltd, USA) then sealed from the external irrigation with some red ribbon wax. The two wires for each thermocouple were connected to a data logger (Pico TC-08 Thermocouple data logger Pico Technology Ltd, UK), which was interfaced to a personal computer. A data acquisition software package (PicoLog) was used to collect the data once per second throughout the osteotomy. A third thermocouple was placed in close proximity to the osteotomy site to record the ambient temperature during the osteotomy preparation.

### Statistical analysis

The data collected were interred into statistical software SPSS® (Ver.20, SPSS Inc, Chicago, IL) for analysis. Descriptive analysis of the data was carried out and maximum temperatures noted. The data were tested for homogeneity of variances using Levene’s test, then data were analyzed using an Independent sample t-test and the significance level was set at P ≤ 0.05.

## Results

**Fig. 4:**
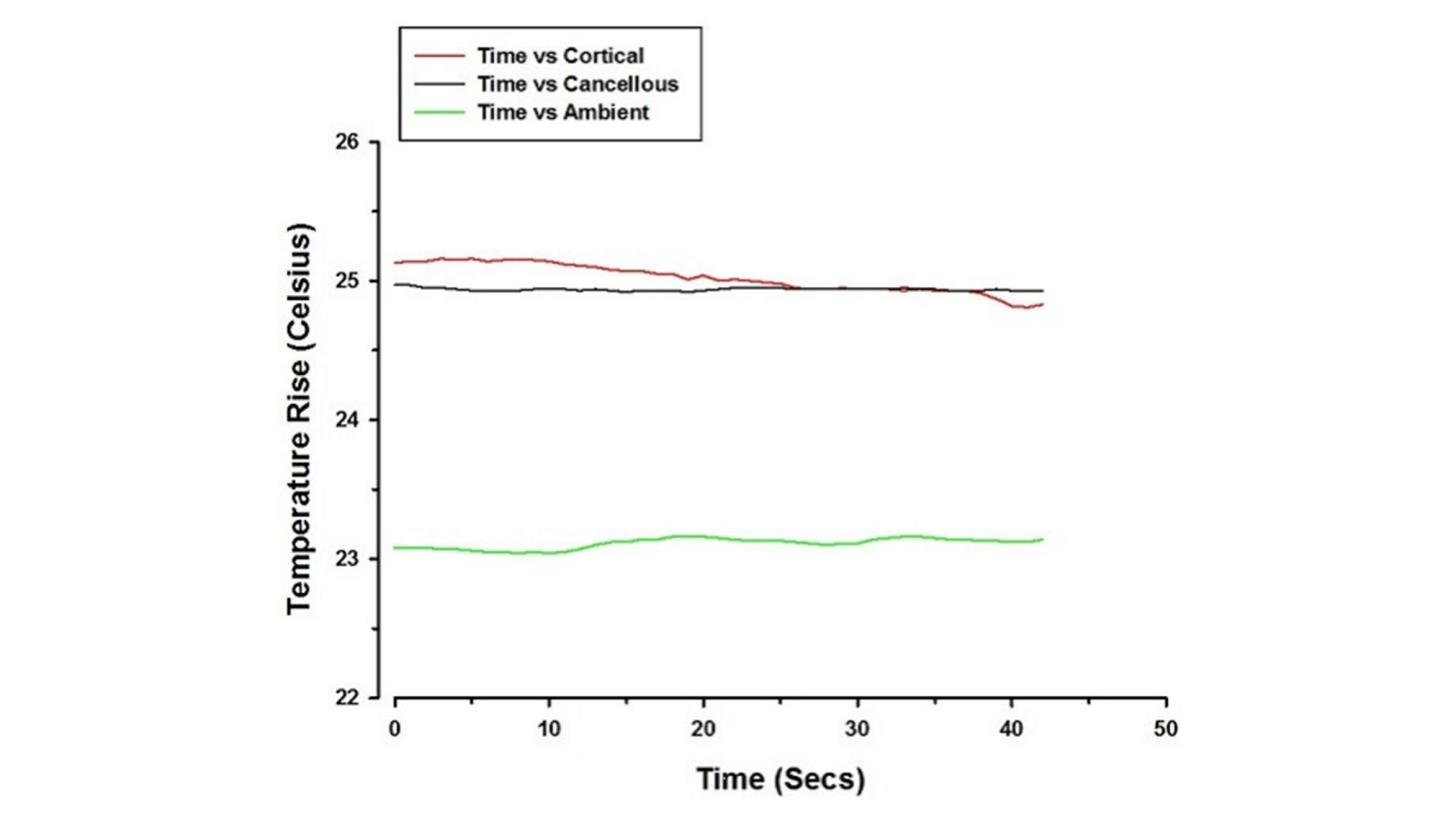
Graph illustrating temperature rise plotted against time for non-limiting surgical guide (Ambient temperature was measured to control the study environment)

**Fig. 5:**
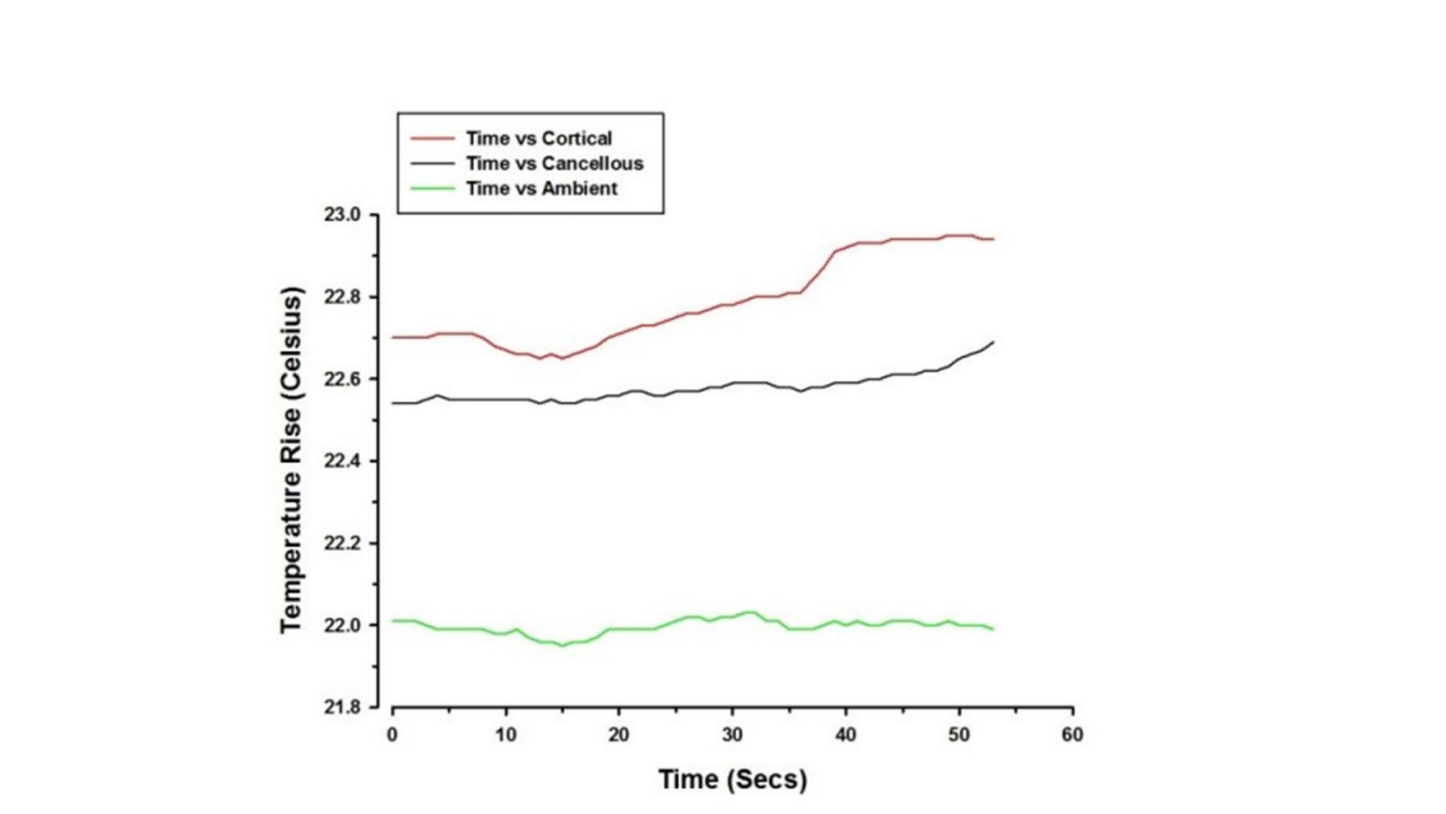
Graph illustrating temperature rise plotted against time for limiting surgical guide (Ambient temperature was measured to control the study environment)

The mean temperature rise for all samples was 0.55 °C **(**Table 1**)**. The mean temperature rises for the limiting surgical guide and non-limiting surgical guide were 0.80 °C and 0.33 °C respectively. Representative graphs of temperature change from the baseline plotted against time for both limiting and non-limiting surgical guides are shown in Figs. 4 and 5. The temperature continued to rise from the baseline for both limiting and non-limiting surgical guides with the limiting surgical guide showing slight oscillation from time to time, due to the pilot drill engaging the metal sleeve, and subsequent variable/intermittent irrigant access.

**Table 1:** The means and standard deviations for both limiting and non-limiting stents at cortical and cancellous bone level

Position	Type of stent	Mean	St. Deviation	P value
Cortical	Limiting	0.82	0.81	0.036
	Non-limiting	0.30	0.73	
	**Total**	**0.54**	**0.80**	
Cancellous	Limiting	0.78	0.88	0.1
	Non-limiting	0.35	0.84	
	**Total**	**0.56**	**0.87**	
Overall	Limiting	0.80	0.84	0.008
	Non-limiting	0.33	0.77	
	**Total**	**0.55**	**0.83**	

*P value of Independent sample t-test

There was a statistically significant difference in temperature rise between the limiting and non-limiting surgical guides (Table 1, P = 0.008), where the limiting surgical guide showed higher readings. In relation to position of temperature recording (coronal vs. apical), there was no statistically significant difference (P ≥ 0.05). No statistically significant difference was noted between the two groups at cancellous bone level (P = 0.68), but the difference was significant at cortical bone level (P = 0.036).

The peak temperature rise for both limiting and non-limiting surgical guides varied between samples. Overall, the peak temperature rise for all samples ranged from 0 to 3.34 °C. None of the samples for both limiting and non-limiting surgical guides exceeded the critical threshold for thermal injury (10 °C). The positions where the maximum temperature rise was recorded also varied between samples. Mainly, the peak temperature rise recorded for both surgical guides was at the 1.5 mm depth coronally in the cortical layer, but some samples showed some deviation, and had the peak temperature rise recorded at 7 mm depth apically in cancellous layer for both surgical guides.

## Discussion

In the current study, in comparison to non-limiting surgical guides, our data showed that limiting surgical guides lead to higher intra-osseous temperature during pilot bone drilling. So, the null hypothesis that there is no difference in temperature changes between limiting and non-limiting surgical guides at different depths was rejected.

It is not surprising that peak temperatures were recorded mostly with a limiting surgical guide compared to the non-limiting one as the metal tube will prevent the irrigant from cooling the drill [17, 18], it is also possible that some frictional heat might be generated as the drill touches the guide during the osteotomy procedure. The difference between non-limiting and limiting surgical guide temperature records was statistical significant, however, all recorded temperatures did not exceed the critical threshold for thermal injury (less than 10 °C rise) [5].

The findings of our study are similar to those that have been reported previously, for both limiting and non-limiting surgical guides [12], however in that study a higher temperature difference for the limiting groups was recorded (2.50 and 2.55 °C), this could be related to the difference in the bone model used where pig bone was considered in that study. Additionally, they used 1,200 rpm rotational speed and 0.5 mm distance between the thermocouple and the osteotomy site compared to 800 rpm rotational speed and 1 mm distance in this study. On the other hand, Jeong et al. reported no significant difference between guided flapless procedures and flap procedures, this difference could be related to the resin models that have been used in this study and the preparation protocol [15].

Some studies have reported higher values, likely due to different drilling speed and bone models [10, 17]. As well as higher temperatures being seen with a limiting static surgical guide, than with a non-limiting guide, higher temperatures have also been demonstrated in high-density bone [16], with cooling of irrigation fluid reported to significantly decrease heat production in osteotomy sites. It should be emphasized that there are significant variations in the bone models (and use of bone versus synthetic substitutes) and experimental setups in the (limited) literature, making direct comparisons with the provided data problematic, however our findings are generally in agreement with previous studies that guided surgery (limiting surgical guides) generates more heat than other drilling techniques, and guides restrict the flow of irrigant [16]. It is worth noting that there are a variety of guide designs available, with some guides being designed to guide the implant drills whilist still allowing coolant access to the osteotomy site (e.g. partially limiting guides), additionally other factors such as use of internally irrigated drills would give differing results. The results in this study relate to preparation of a pilot osteotomy, it would be expected that use of larger drills (with increased diameter/surface area) would result in higher temperature increase, especially if used in a fully-guided protocol with a fully limiting guide.

Although there was a difference in temperature rise between cortical and cancellous layers it is not statistically significant nor of any clinical relevance. Most of the peak temperatures were recorded with a limiting surgical guide coronally, which could be related to the density of the bone coronally, as the harder the bone, the more heat is generated, with greater torque and power needed [16, 19]. With deeper penetration of the drill, heat loss is reduced and there is further inability of irrigant to reach deeper layers, which may contribute to comparable values coronally and apically despite softer bone apically. Previous research has shown that as drilling depth rises, external irrigation does not offer enough bone cooling [20, 21]. Because human blood circulation within the bone allows for some degree of cooling during procedures, in vitro research utilizing artificial bone models or cadavers encounter limitations in recreating this cooling mechanism [22]. As a result, the real temperature in clinical circumstances may be lower and less harmful to bone structures [23]. It is also worth noting that temperatures recorded in bovine bone have been shown to be greater than that of the human bone, attributed to a variation in the density of cortical bone and the effect of blood flow on dissipating heat [19].

Bovine ribs have been used for previous in vitro studies, suggesting they are suitable to compare to human tissues [24–26], and was selected for the study because it was readily available, is isotropic and has comparable levels of thermal conductivity and density to human bone (although not the same) [27, 28].

## Limitations

Although careful selection of bone specimens was undertaken to ensure they were at least 11 mm in thickness and had sufficient amount of cortical and cancellous layers in order to reduce the variability between the samples, some specimens had very dense bone in comparison to others, which could not be avoided. This might have introduced some variability in the results, consequently having very high values of peak temperatures. As the harder the bone, the more heat is generated, due to the differences in torque and power, thrust is needed. Random assigning of bone specimens to each surgical guide type was intended to reduce any variability in results. Moreover, with the limiting surgical guide the metal sleeve did not allow the bur to go further than 9 mm as the head of the contra angle was touching the guide sleeve at the 9 mm depth. This could have been avoided if a longer pilot drill was used, or by reducing the metal sleeve height. Even then, the thickness of the bovine ribs might not have allowed the drill to reach the standard implant length. Therefore, the depth of the osteotomy was decided to be 9 mm due to the limited thickness of the bovine ribs.

## Conclusions

Within the limitations of this study, the following conclusions may be drawn:-.

1. When using surgical guides for pilot implant bed preparation heat will be generated.
2. Generated heat with pilot drills used with non-limiting or limiting surgical guides results in temperatures below the thermal necrosis limit.
3. Limiting surgical guides will generate more heat using pilot drills than non-limiting surgical guides; however, the amount of heat generated is not of any clinical importance and within the safe range.
4. The effect of heat generated for both surgical guides is different at different depths.

## Data Availability

All collected data analyzed during this study are included in this published article. Some datasets are available from the corresponding author on reasonable request.

## Abbreviations

Cº: Degree Celsius
SPSS: Statistical Package for the Social Sciences
TC: Thermocouple
